# Identification of a signature of histone modifiers in kidney renal clear cell carcinoma

**DOI:** 10.18632/aging.205944

**Published:** 2024-06-17

**Authors:** Yongming Huang, Zhongsheng Yang, Ying Tang, Hua Chen, Tairong Liu, Guanghua Peng, Xin Huang, Xiaolong He, Ming Mei, Chuance Du

**Affiliations:** 1 Department of Urology, Ganzhou People's Hospital, Ganzhou, Jiangxi 341000, China; 2 Department of Day Ward, The First Affiliated Hospital, Jiangxi Medical College, Nanchang University, Nanchang, Jiangxi 330006, China

**Keywords:** kidney renal clear cell carcinoma, histone modifiers, risk score, prognosis

## Abstract

Kidney renal clear cell carcinoma (KIRC) is a cancer that is closely associated with epigenetic alterations, and histone modifiers (HMs) are closely related to epigenetic regulation. Therefore, this study aimed to comprehensively explore the function and prognostic value of HMs-based signature in KIRC. HMs were first obtained from top journal. Then, the mRNA expression profiles and clinical information in KIRC samples were downloaded from The Cancer Genome Atlas (TCGA) database and Gene Expression Omnibus (GEO) datasets. Cox regression analysis and least absolute shrinkage and selection operator (Lasso) analysis were implemented to find prognosis-related HMs and construct a risk model related to the prognosis in KIRC. Kaplan-Meier analysis was used to determine prognostic differences between high- and low-risk groups. Immune infiltration and drug sensitivity analysis were also performed between high- and low-risk groups. Eventually, 8 HMs were successfully identified for the construction of a risk model in KIRC. The results of the correlation analysis between risk signature and the prognosis showed HMs-based signature has good prognostic value in KIRC. Results of immune analysis of risk models showed there were significant differences in the level of immune cell infiltration and expression of immune checkpoints between high- and low-risk groups. The results of the drug sensitivity analysis showed that the high-risk group was more sensitive to several chemotherapeutic agents such as Sunitinib, Tipifarnib, Nilotinib and Bosutinib than the low-risk group. In conclusion, we successfully constructed HMs-based prognostic signature that can predict the prognosis of KIRC.

## INTRODUCTION

Hundreds of thousands of people are affected by renal cell carcinoma (RCC) every year around the world [1]. According to its pathological characteristics, RCC can be divided into various subtypes, among which the kidney renal clear cell carcinoma (KIRC) is the most common subtype, accounting for more than 70% of all cancers [2]. Early stage KIRC is insidious and patients have no obvious symptoms, so it often leads to the delay in diagnosis and treatment [3]. In addition, about 1/3 of early stage KIRC can eventually develop metastasis [4]. These characteristics of KIRC led to the dilemma of unsatisfactory prognosis in KIRC. Therefore, it makes sense to identify meaningful biomarkers of KIRC to ameliorate this dilemma. Many studies have shown that multi-gene signature allows for more accurate risk assessment and prognostic prediction of cancers [5–8]. Therefore, in this study, a risk model of HMs-based signature in KIRC was established to predict the prognosis of KIRC.

Histone modifiers (HMs) can code and decode modifications of histone residues and are usually further divided into readers, writers and erasers [9]. The readers usually contain specific domains that identify histone residues and determine their modification types and states, and writers and erasers often play a role in adding and removing modifications to certain histone residues, such as acetylation and deacetylation, methylation and demethylation, ubiquitination and deubiquitination, etc., [10–12]. In addition to being associated with histone modifications, HMs can also interact with transcription factors or other proteins to form a complex regulatory network that ultimately results in a sophisticated regulation of gene expression [12]. Although research on HMs has been conducted for decades, and some results have been achieved in recent years [13–17], a comprehensive analysis of the function and its prognostic value of HMs in KIRC remains lacking. A focus was placed on function and prognostic value of HMs in KIRC in the present study. We constructed a risk model for the KIRC samples consisting of the expression level of 8 HMs (*C17orf49, GLYATL1, HJURP, KAT2A, NCOA7, NEK6, PRDM16, TTK*). We examined the correlation between HMs-based signature and clinical characteristics of KIRC samples and constructed a signature based on HMs that could be used to predict prognosis of KIRC. To further explore the molecular mechanisms of the signature, we also investigated differences in the immune characteristics of KIRC samples in the groups at high and low risk. We finally also analyzed the sensitivity of KIRC samples to chemotherapy drugs between the groups at high and low risk to evaluate the value of the clinical application of the signature.

## METHODS

### Data acquisition and acquisition of HMs

MRNA expression profiles, clinicopathological data and prognostic information derived from 539 KIRC samples and 72 normal control samples were obtained from The Cancer Genome Atlas (TCGA, https://portal.gdc.cancer.gov/) database [18]. A total of 540 histone modifiers (HMs) were derived from the top published journals [19].

### Differential expression analysis and functional enrichment analysis of HMs

Differentially expressed HMs in KIRC samples and normal control samples were screened out based on the screening criteria of |logFC| >1 and false discovery rate (FDR) <0.01 by applying the limma R package. To mine the underlying molecular mechanisms of HMs, GO (Gene Ontology) and KEGG (Kyoto Encyclopedia of Genes and Genomes) pathway enrichment analyses were implemented by running R.

### Cluster analysis

To explore the relationship between the expression of differentially expressed HMs and the KIRC, consensus clustering analysis was performed in KIRC by applying the limma R package and ConsensusClusterPlus R package. In addition, survival differences between different clusters and clinical characteristics were further analyzed by using the survival, survminer and pheatmap R packages.

### Construction and validation of a risk model of HMs-based signature

KIRC samples applied in the construction of the risk model must meet the following criteria: (1) samples have full expression information and (2) the overall survival time of the samples were more than 30 days from the time of diagnosis of KIRC. Then, by applying the caret R package the samples meeting the inclusion criteria were divided into a training set and a test set in a ratio of 7:3 according to the caret sampling method.

Univariate analysis was implemented to identify HMs associated with the prognosis of KIRC in the training set by running R. Then, least absolute shrinkage and selection operator (LASSO), a regularization method in regression analysis, was implemented to eliminate HMs that were overfitted with the model by applying the glmnet R package. The risk score in the model was estimated by the following formula: Risk score = (Coef1 × expression of gene1) + (Coef2 × expression of gene2) + … + (Coef n × expression of gene3). Based on the median risk score of this model, the KIRC samples in the training set can be categorised into high- and low-risk groups. Principal component analysis (PCA) and t-distributed stochastic neighbor embedding (t-SNE) were used to downscale the expression of all genes in the model. The Kaplan–Meier survival curve and receiver operating characteristic (ROC) curve of HMs-based signature were plotted to display the model’s ability to predict the prognosis of the sample by running R. The test set and the entire set were implemented with the same procedures to examine reliability of this risk model constructed from the samples in the training set.

### Construction of the nomogram

The relationship between clinical variables and HMs-based signature was explored. Univariate and multivariate analyses were implemented to find independent prognostic factors for KIRC samples. Then, independent prognostic factors were included into the establishment of nomogram which could forecast 3-year and 5-year overall survival (OS) in KIRC, and the calibration curves visualising the comparison between the 3-year and 5-year survival probabilities forecasted by the nomogram for KIRC and the observed actual survival probabilities were then plotted.

### Immune characteristics of the HMs-based signature

KIRC is an immunogenic tumor and its development and progression are associated with immune characteristics in the tumor microenvironment. Based on this basis, the relationship between the expression level of 8 HMs in the risk model and the level of immune cell infiltration was analysed in the TIMER database (https://cistrome.shinyapps.io/timer/) [20]. The relationship of HMs with microsatellite instability (MSI) and tumor mutation burden (TMB) was further analyzed given that MSI and TMB can respond to immunotherapy of tumors. Then, the differences in tumor microenvironment (TME) between high- and low-risk groups in the risk model were explored. Since the level of immune checkpoints can reflect the immune status in tumor, the characteristics of expression of immune checkpoints between high- and low-risk groups were further explored. A major cause of immunotherapy failure and tumor progression is immune escape. The tumor immune dysfunction and exclusion (TIDE, http://tide.dfci.harvard.edu/) [21] score is a good indicator to evaluate tumor immune escape [21]. Therefore, differences in TIDE scores between high- and low-risk groups were analyzed to assess the probability of immune escape in different KIRC samples.

### Correlation between drug sensitivity and risk signature

To investigate the sensitivity of KIRC samples to common chemotherapy drugs between different risk groups, the Genomics of Drug Sensitivity in Cancer (GDSC, https://www.cancerrxgene.org/) database [22] and the pRRophetic R package were applied to analyze differences in IC_50_ of KIRC samples in chemotherapy drugs between different risk groups.

### Statistics analysis

All statistical analyses were implemented by R (version 4.0.0). Wilcoxon Rank-Sum test was implemented to compare gene expression differences between KIRC samples and control samples. Pearson’s correlation coefficient was applied to estimate the linear relationship between the variables. *P*-values less than 0.05 were considered significant differences.

### Availability of data and materials

Raw data in this study can be obtained from the GDC database (https://portal.gdc.cancer.gov/), TIDE database (http://tide.dfci.harvard.edu/) and GDSC database (http://www.cancerrxgene.org/).

## RESULTS

### Identification and functional enrichment of differentially expressed HMs

**Figure 1:**
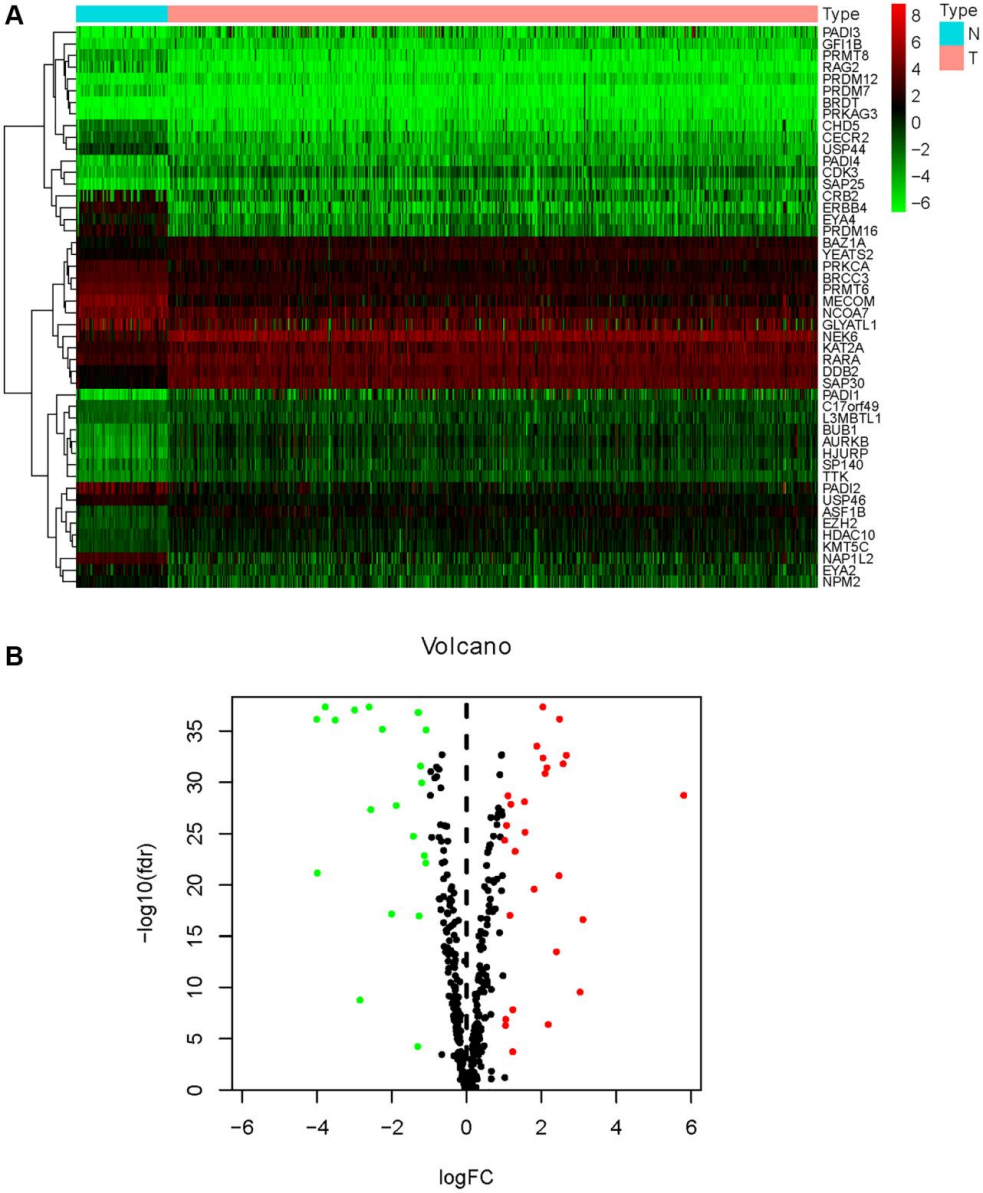
**Differential expression analysis of HMs.** (**A**) The heatmap displayed the differential expression of HMs between KIRC samples and normal control samples. Red represented up-regulation of HMs expression, green represented down-regulation of HMs expression. (**B**) Volcano displayed the differential expression of HMs in KIRC samples.

**Figure 2:**
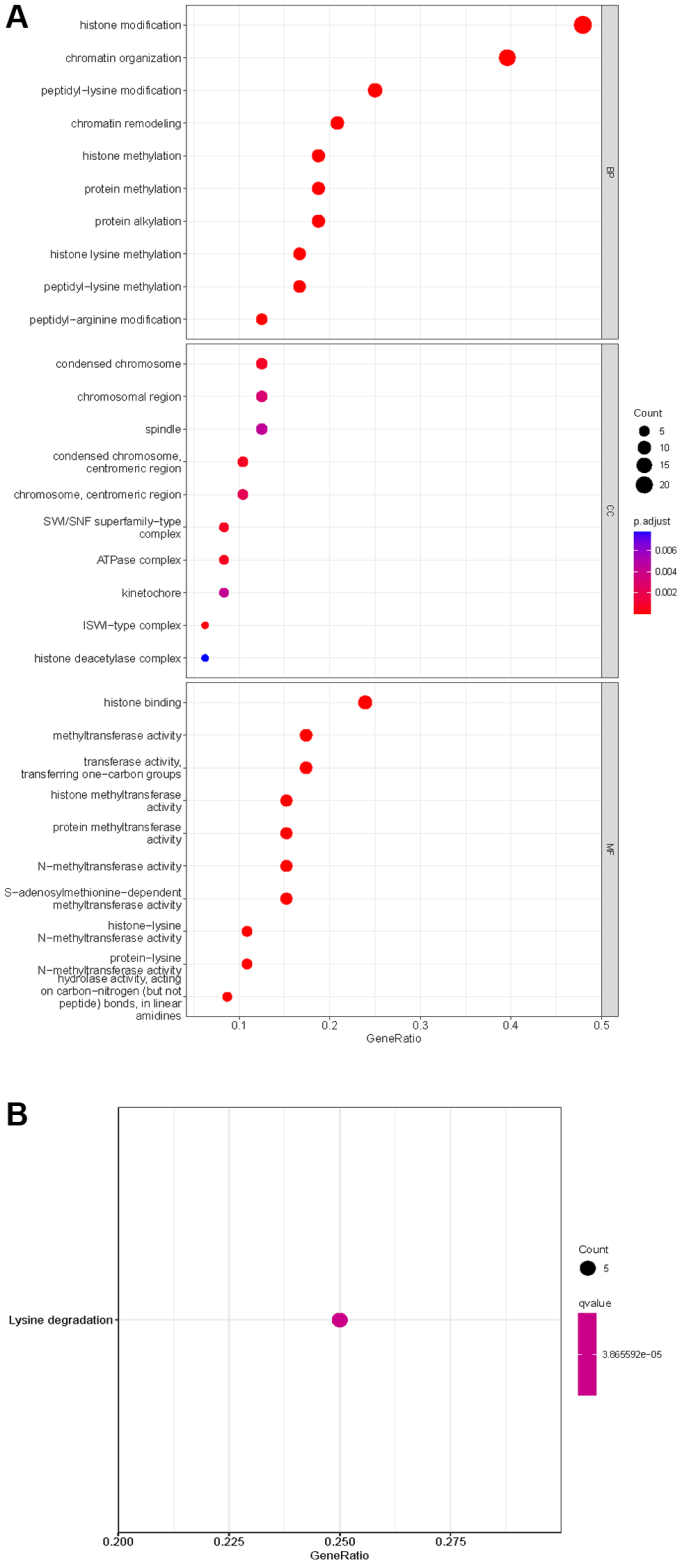
**Enrichment analysis of HMs differentially expressed in KIRC.** (**A**) GO analysis of HMs. (**B**) KEGG pathway enrichment analysis of HMs.

Of the 539 KIRC samples and 72 control normal samples, 21 down-regulated and 27 up-regulated HMs were identified and the results were presented in Figure 1A, 1B. GO analysis (Figure 2A) showed that the GO terms with the highest enrichment in the biological process (BP) classification were histone modification, chromatin organization, peptidyl−lysine modification, chromatin remodeling and histone methylation. In the cellular component (CC) content, condensed chromosome, chromosomal region and spindle were the main enriched GO terms. Histone binding, methyltransferase activity, transferase activity and transferring one−carbon groups were the significantly enriched GO terms in the category of molecular function (MF). The enrichment analysis of KEGG pathways implied that HMs may be closely associated with Lysine degradation (Figure 2B).

### Classification of KIRC samples-based HMs signature

**Figure 3:**
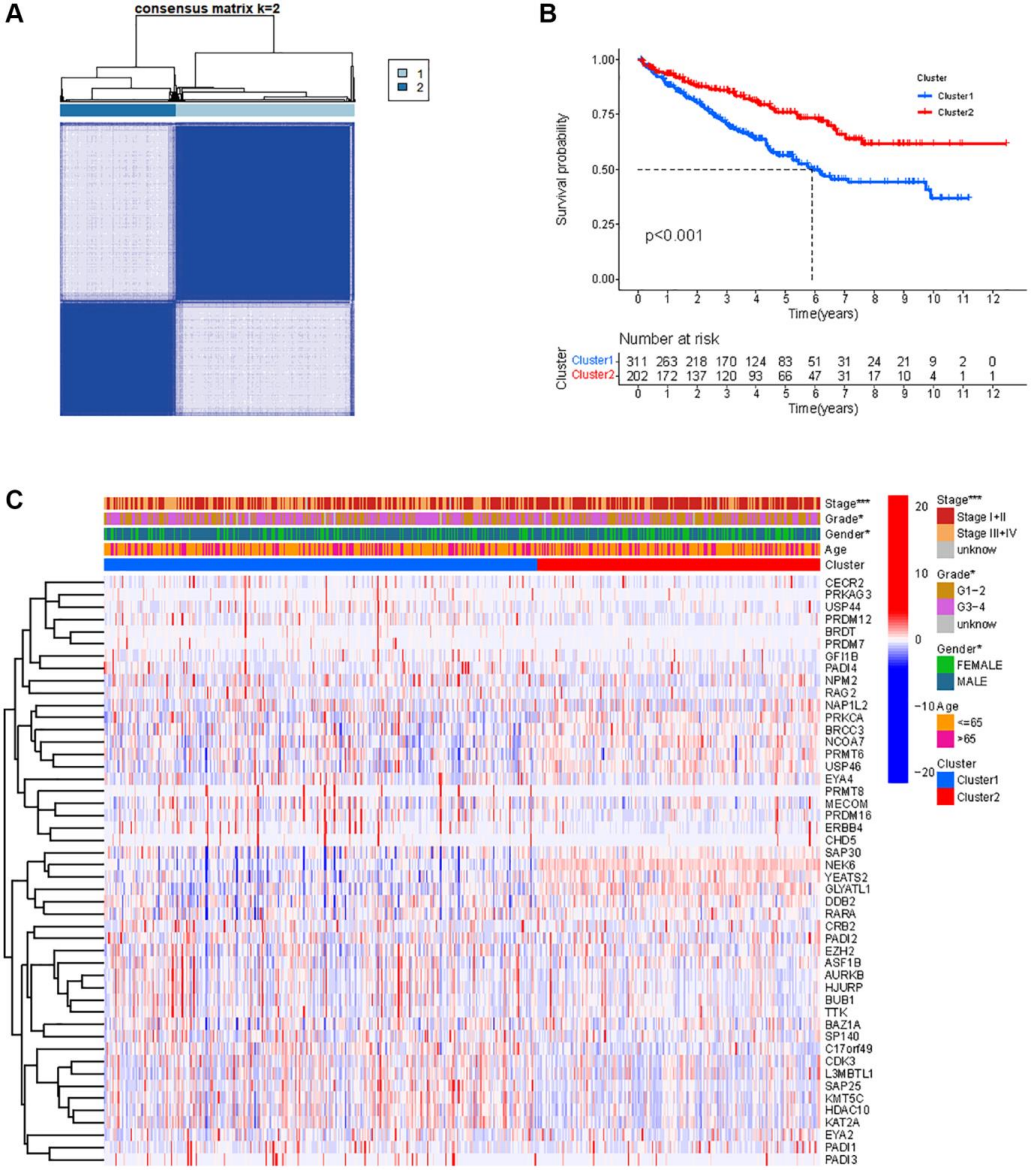
**Classification of KIRC samples based on HMs.** (**A**) 515 KIRC samples can be divided into two clusters by the consensus clustering analysis (k = 2). (**B**) Kaplan-Meier OS analysis for two clusters. (**C**) Heatmap of the expression of HMs and clinical characteristics of the KIRC samples in two clusters.

When performing consensus cluster analyses on the KIRC samples by clustering variable (k) from 1 to 9, we found that the strongest correlations between samples within groups were found when k = 2 (Figure 3A). Subsequently, the 515 KIRC samples were divided into two clusters. Kaplan-meier (KM) survival analysis between the two clusters showed that OS of KIRC samples in cluster 1 was significantly worse than OS in cluster 2 (*p* < 0.001) (Figure 3B). We present a heat map depicting the association between the mRNA expression of HMs and the clinical characteristics of KIRC samples, including Age, Gender, Grade and Stage (Figure 3C). We found significant differences between the KIRC samples in Cluster 1 and Cluster 2 in terms of Gender (*p* < 0.05), stage (*p* < 0.001) and grade (*p* < 0.05) of the tumor.

### Construction and verification of HMs-based signature

**Figure 4:**
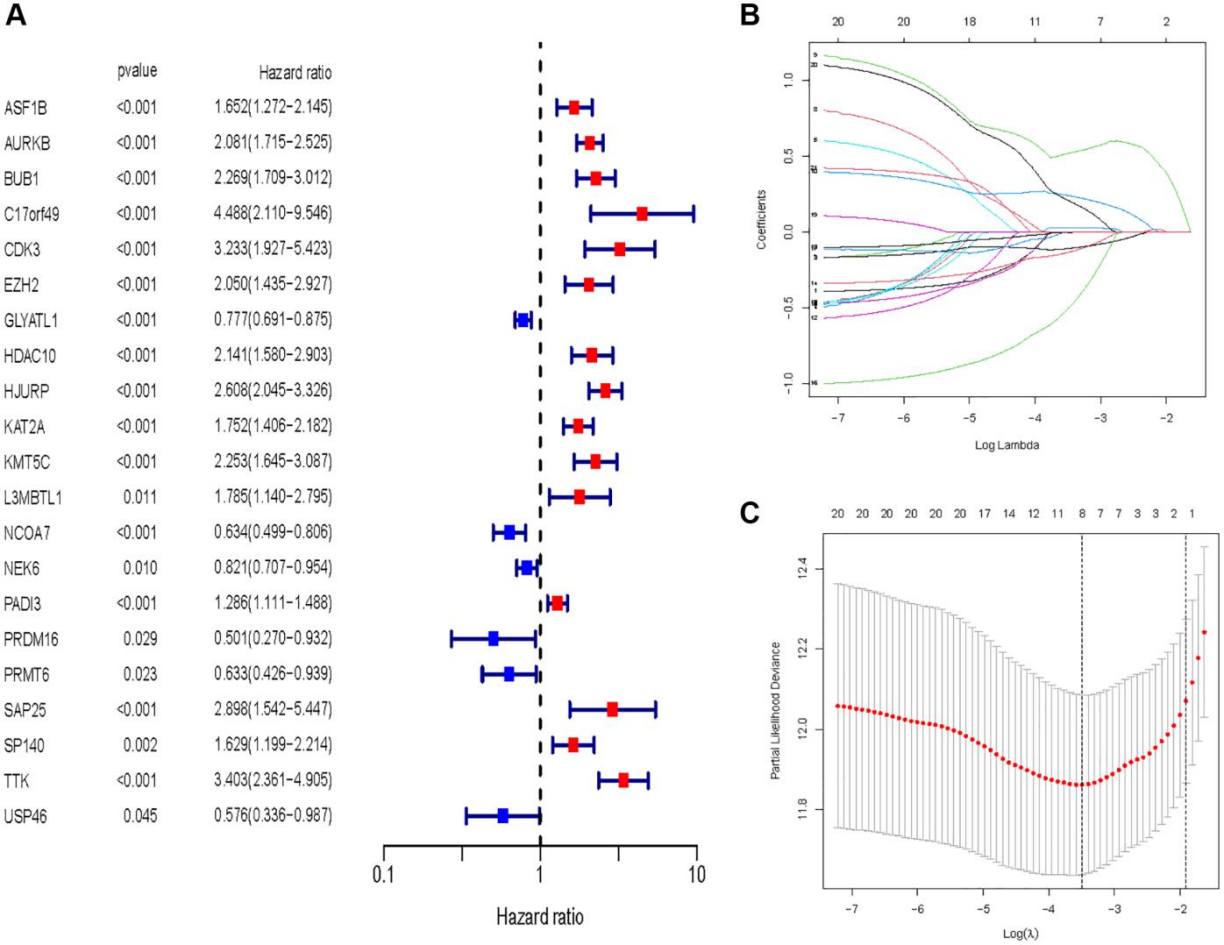
**Identification of HMs for construction of risk model.** (**A**) Identification of differentially expressed HMs associated with prognosis of KIRC samples by univariate analysis. (**B**) Elimination of HMs with model overfitting by LASSO analysis. (**C**) Tenfold cross-validation for tuning parameter selection in the LASSO analysis.

**Figure 5:**
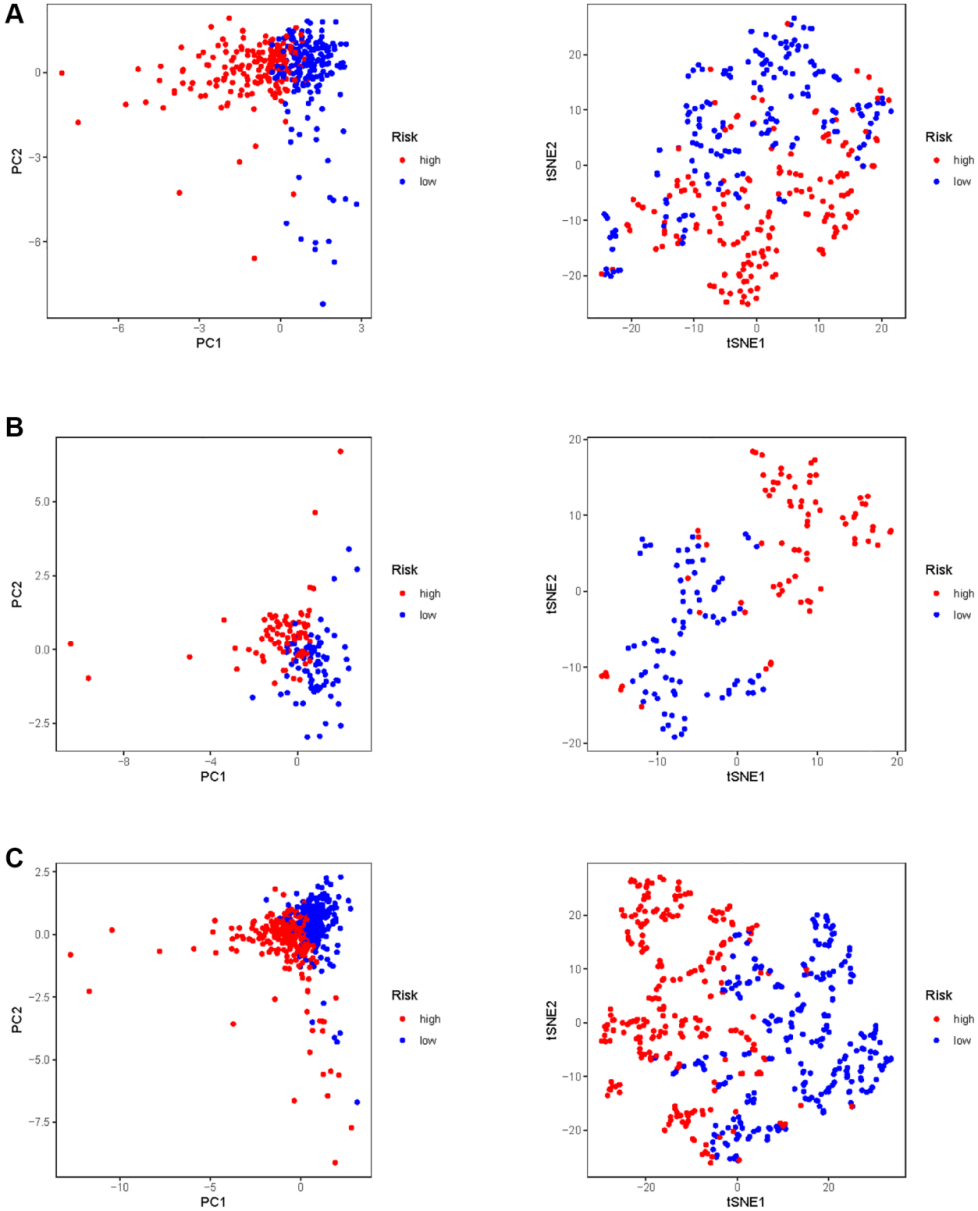
**PCA and t-SNE analysis of risk model in KIRC.** (**A**) PCA and t-SNE analysis of risk model in training set. (**B**) PCA and t-SNE analysis of risk model in test set. (**C**) PCA and t-SNE analysis of risk models in entire set.

**Figure 6:**
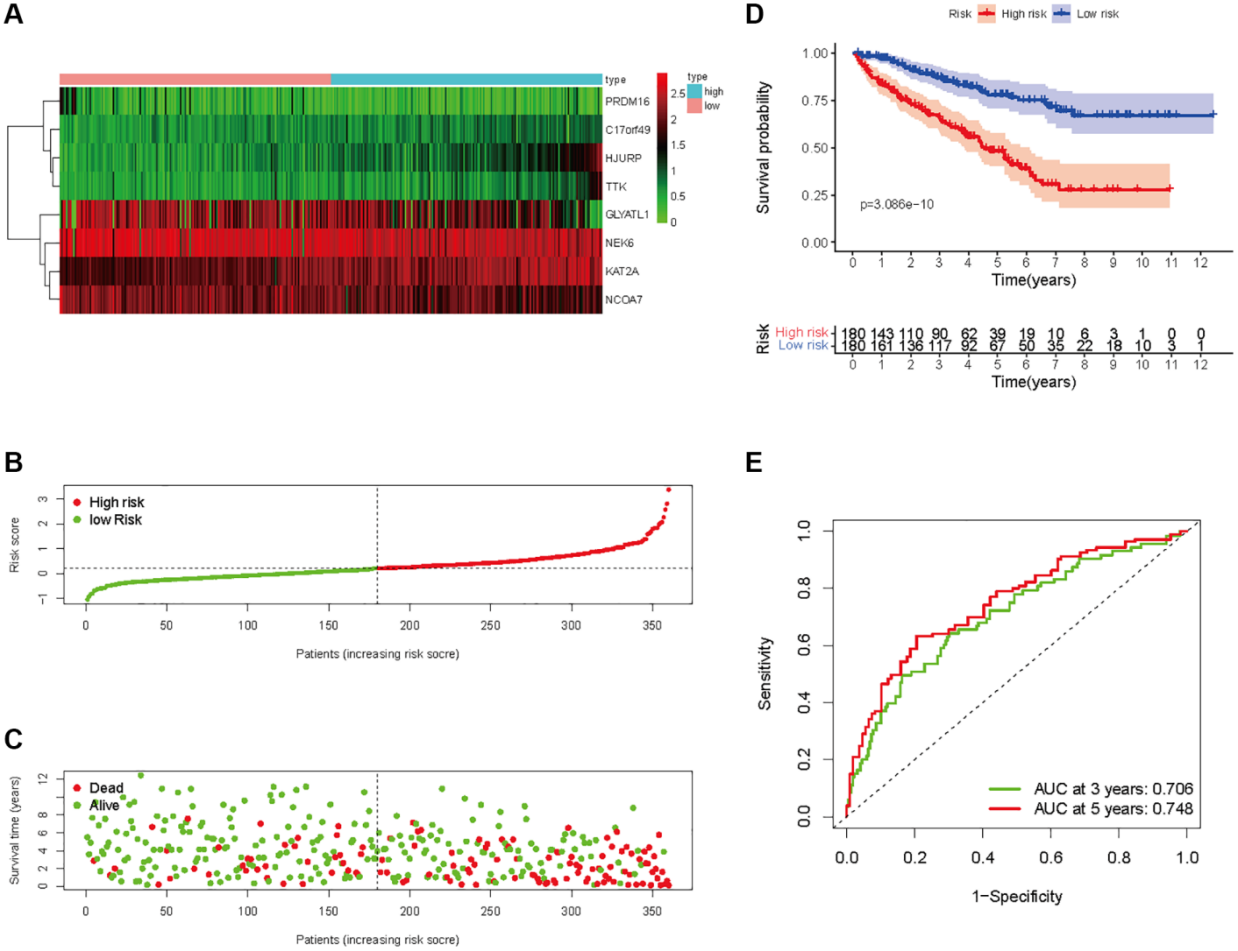
**Prognostic analysis of HMs-based signature in the training set.** (**A**) Expression of HMs of KIRC samples between high- and low-risk groups. (**B**) Kaplan-Meier survival analysis of KIRC samples between high- and low-risk groups. (**C**, **D**) Analysis of differences in survival status of KIRC samples between high- and low-risk groups. (**E**) Time-dependent ROC curve analysis of risk scores predicting 3- and 5- year OS in the KIRC samples.

**Figure 7:**
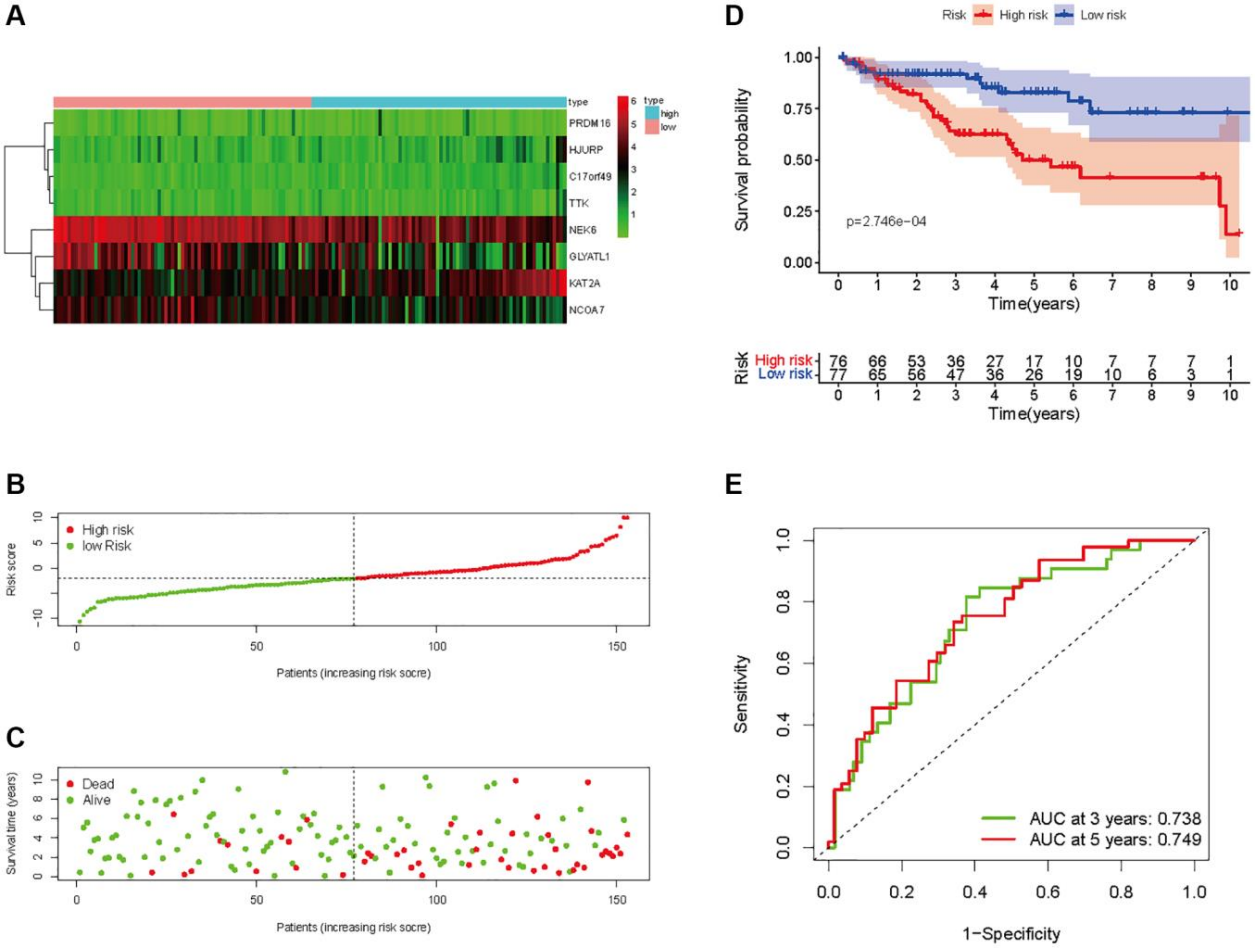
**Prognostic analysis of HMs-based signature in the test set.** (**A**) Expression of HMs of KIRC samples between high- and low-risk groups. (**B**) Kaplan-Meier survival analysis of KIRC samples between high- and low-risk groups. (**C**, **D**) Analysis of differences in survival status of KIRC samples between high- and low-risk groups. (**E**) Time-dependent ROC curve analysis of risk scores predicting 3- and 5-year OS in the KIRC samples.

**Figure 8:**
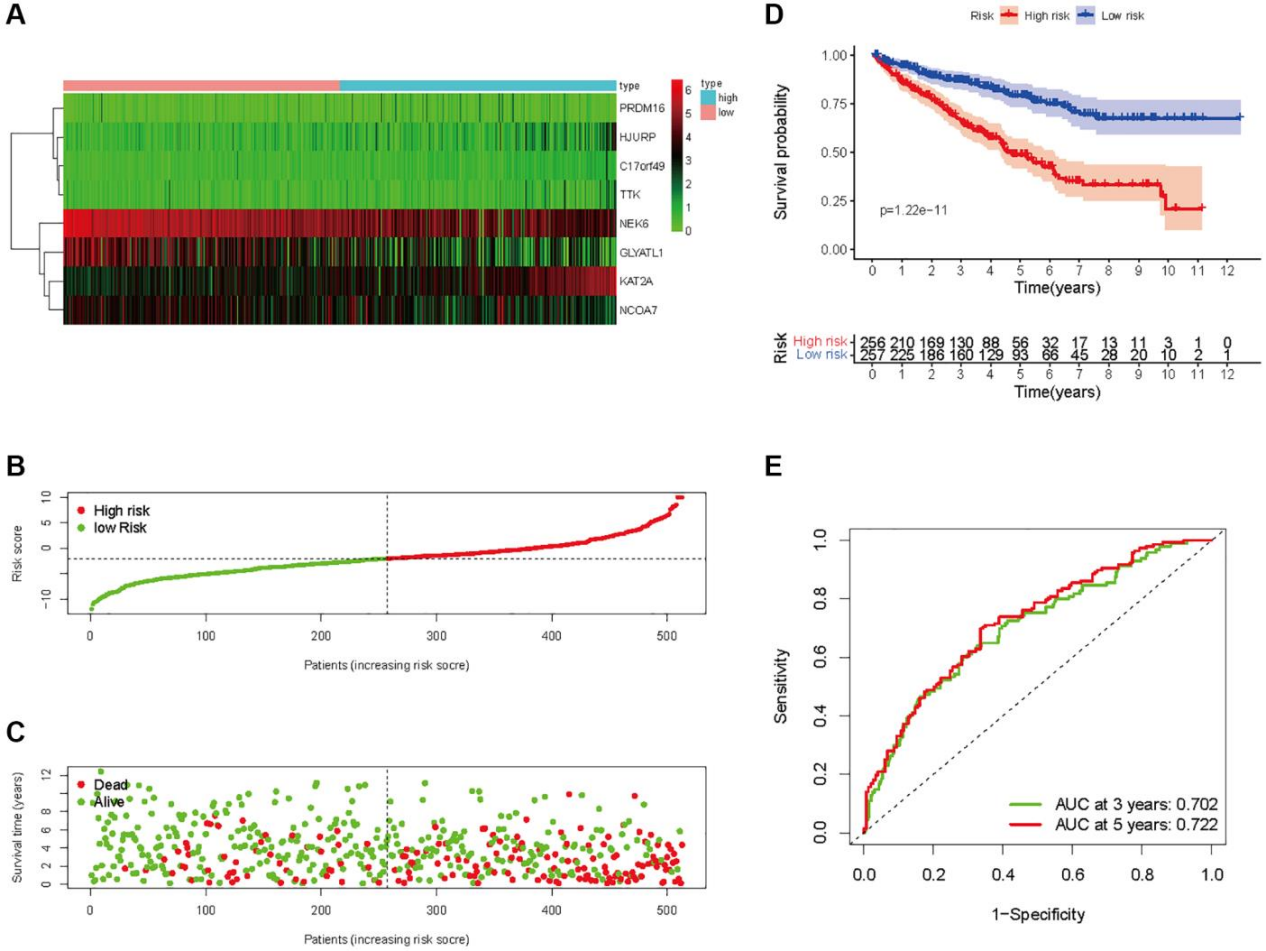
**Prognostic analysis of HMs-based signature in the entire set.** (**A**) Expression of HMs of KIRC samples between high- and low-risk groups. (**B**) Kaplan-Meier survival analysis of KIRC samples between high- and low-risk groups. (**C**, **D**) Analysis of differences in survival status of KIRC samples between high- and low-risk groups. (**E**) Time-dependent ROC curve analysis of risk scores predicting 3- and 5-year OS in the KIRC samples.

In all, 515 samples from the KIRC met the inclusion criteria for follow-up research. These KIRC samples were further divided into 361 samples as a training set and 154 samples as a test set in a 7:3 ratio by care method sampling. Univariate analysis of differentially expressed HMs in the training set were performed and 21 HMs that significantly correlated with the prognosis of KIRC were identified (Figure 4A). Those HMs that were over-fitted to the model were eliminated, and finally 8 HMs (*C17orf49, GLYATL1, HJURP, KAT2A, NCOA7, NEK6, PRDM16, TTK*) were identified to build a risk model (Figure 4B, 4C). The risk score can be calculated from the mRNA expression of HMs and the relevant coefficients (Table 1) with the following equation: riskscore = 0.0280971627824297 × *C17orf49* expression + (−0.107290283832969) × *GLYATL1* expression + 0.520165069344535 × *HJURP* expression + 0.241951166184651 × *KAT2A* expression + (−0.00243111668616741) × *NCOA7* expression + (−0.13291750024586) × *NEK6* expression + (−0.500207346820667) × *PRDM16* expression + 0.209483791892391 × *TTK* expression. As a cut-off value, the median risk score was used to divide the KIRC samples into high-risk and low-risk categories. In the two-dimensional plane, PCA and t-SNE analysis (Figure 5) showed significant dispersion between KIRC samples of high- and low-risk groups. The mRNA expression of the 8 HMs in the high-risk group and the low-risk group was shown in Figure 6A. The correlation between the risk signature and the survival of the KIRC samples were shown in Figure 6B–6D. The KIRC samples in the high-risk group had significantly shorter survival time and significantly higher mortality rates than the samples in the low-risk group. The ROC curve showed an accuracy of 0.706 and 0.748 for the risk score to predict 3- and 5-year OS for the KIRC samples, respectively (Figure 6E). A same analysis was performed on the test set as well as the entire set to verify the stability and reliability of the risk model derived from training set. As shown in Figures 7A, 8A, test and overall sets of KIRC samples of high- and low-risk groups displayed similar expression level of HMs as the training samples. Survival differences between high-risk group and low-risk group in the test (Figure 7B–7D) and overall set (Figure 8B–8D) were consistent with those in the risk model. The accuracy of the test set’s risk score in predicting 3- and 5-year OS for KIRC samples was 0.738 and 0.749, respectively (Figure 7E) and 0.702 and 0.722, respectively in the entire set (Figure 8E). Analyses of the test set and the total set showed good stability and reliability of the risk model.

**Table 1:** Genes and coefficient in risk models.

**Gene symbol**	**Coefficient**
C17orf49	0.028097
GLYATL1	−0.10729
HJURP	0.520165
KAT2A	0.241951
NCOA7	−0.00243
NEK6	−0.13292
PRDM16	−0.50021
TTK	0.209484

### Relationship between HMs-based signature and clinical variables

**Figure 9:**
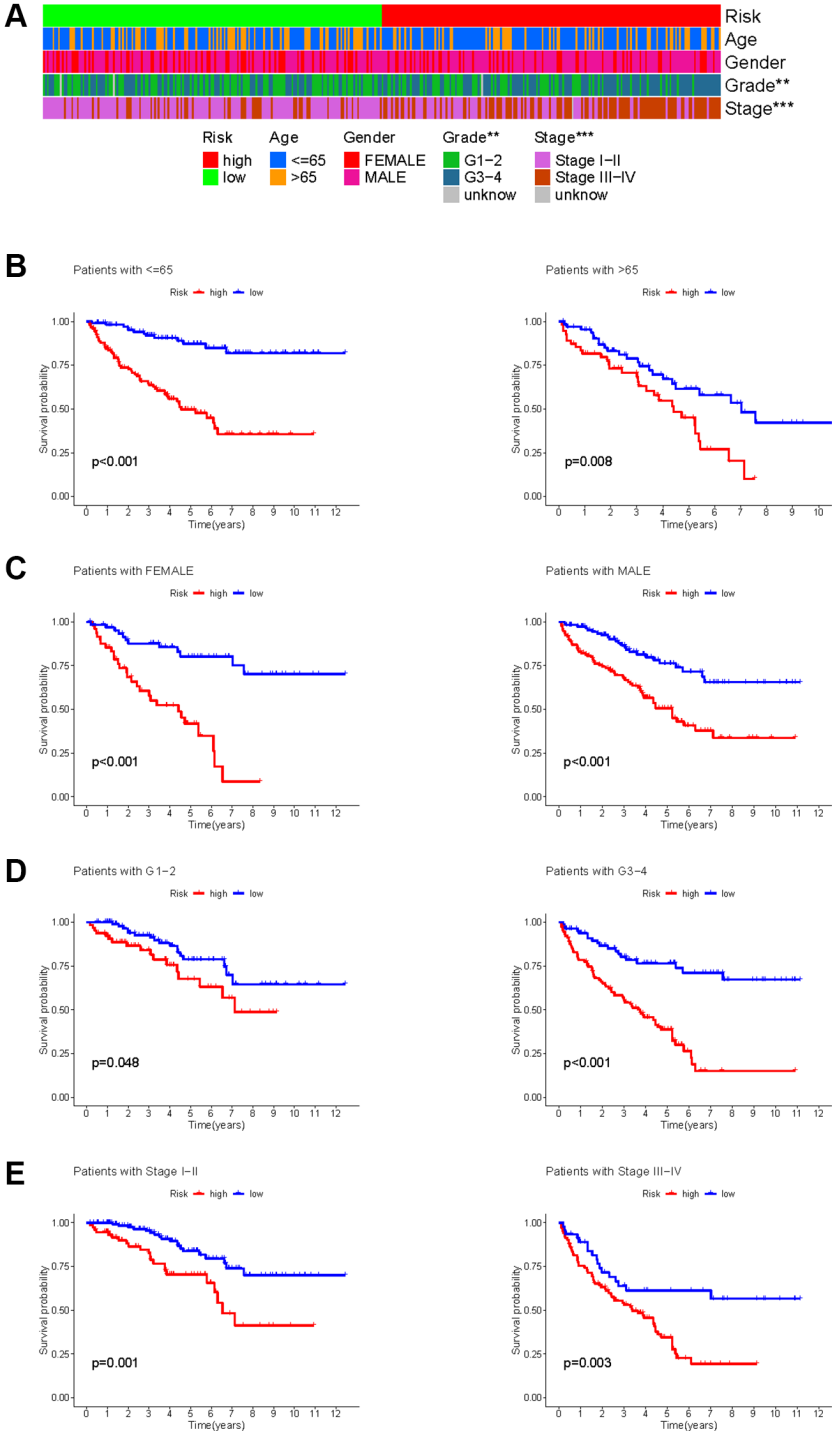
**Correlation between HMs-based signature and clinical characteristics and prognosis.** (**A**) Analysis of differences in clinical characteristics (age, gender, grade and stage) of KIRC samples between high- and low-risk groups. (**B**) Analysis of survival differences between high- and low-risk KIRC samples from subgroup stratified by age (≤60 years and >60 years). (**C**) Analysis of survival differences between high- and low-risk KIRC samples from subgroup stratified by gender (FEMALE and MALE). (**D**) Analysis of survival differences between high- and low-risk KIRC samples from subgroup stratified by grade (G1-2 and G3-4). (**E**) Analysis of survival differences between high- and low-risk KIRC samples from subgroup stratified by stage (Stage I−II and Stage III−IV).

The KIRC samples in the risk model can be divided into subgroups based on age, gender, grade, and stage. The heatmap displayed a significant difference in grade (*p* < 0.01) and stage (*p* < 0.001) between high- and low-risk groups (Figure 9A). The KM survival curves in each subgroup (age ≤65 or >65, gender = FEMALE or MALE, grade = G1−2 or G3−4, stage = I−II or III−IV) showed that each subgroup of KIRC samples with a high risk score had a worse OS than those with a low risk score (Figure 9B–9E).

### Construction of prognostic models

**Figure 10:**
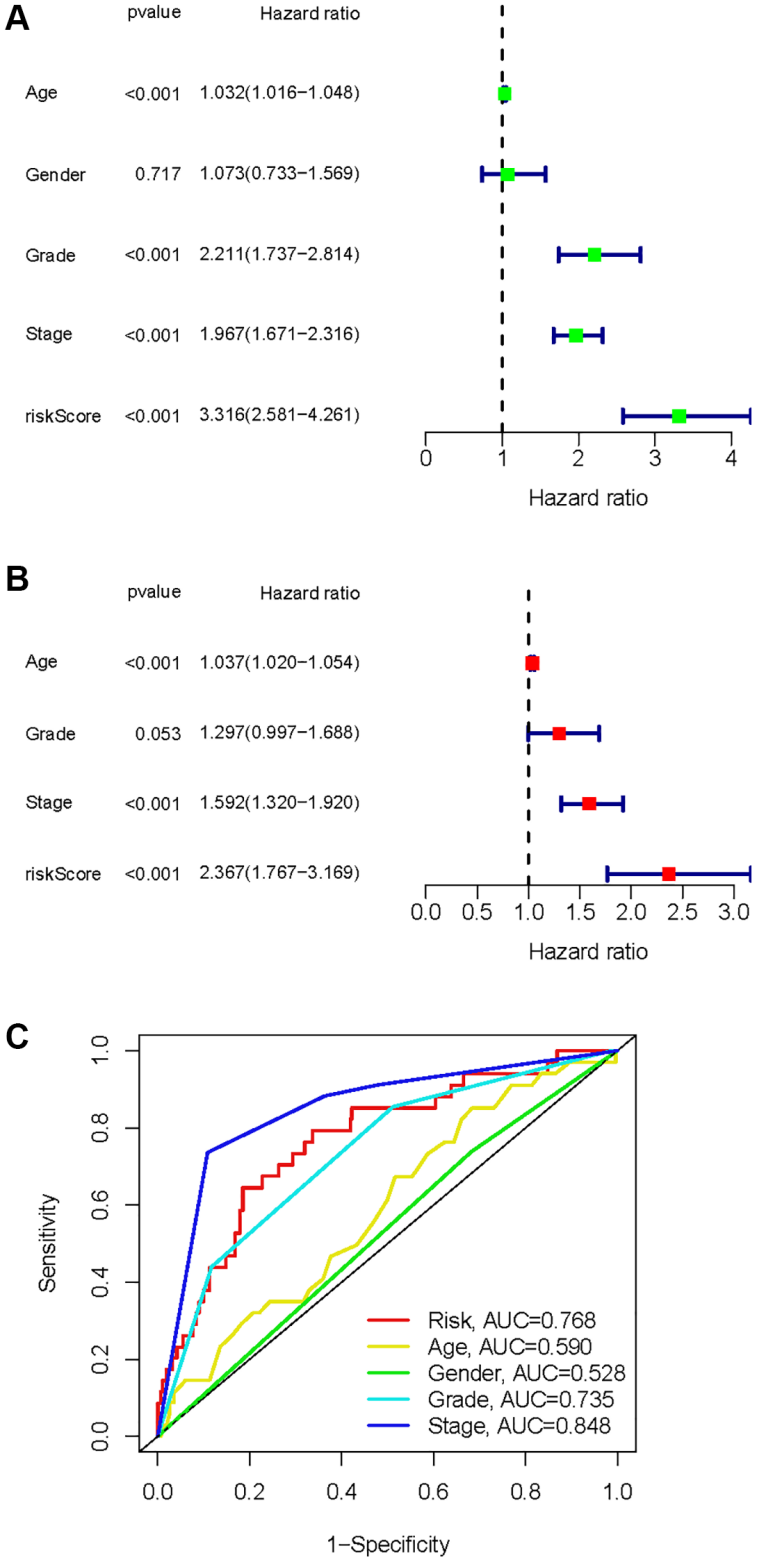
**Identification of independent factors affecting the prognosis of the KIRC samples.** (**A**) Univariate Cox regression analysis to identify factors associated with prognosis in the KIRC sample. (**B**) Multivariate Cox regression analysis to identify factors that can independently predict prognosis in the KIRC sample. (**C**) Multivariate ROC curves for risk and other clinical variables predicting prognosis in the KIRC samples.

A univariate Cox analysis was conducted using variables including age, gender, grade, stage, and risk score (Figure 10A). Univariate Cox analysis revealed close correlations between age (*p* < 0.001), grade (*p* < 0.001), stage and risk score (*p* < 0.001) for the prognosis of KIRC samples. Further multivariate Cox analyses were implemented using age, grade, stage, and risk score. Cox analysis of the KIRC samples revealed that age (*p* < 0.001), grade (*p* = 0.053), stage (*p* < 0.001), and risk score (*p* < 0.001) influenced prognosis independently (Figure 10B). In addition, ROC curves of several clinical variables and risk score predicting the OS of KIRC samples shown that risk score (AUC = 0.768) can be as accurate as or better than some traditional clinical variables in predicting KIRC samples’ prognosis (Figure 10C).

**Figure 11:**
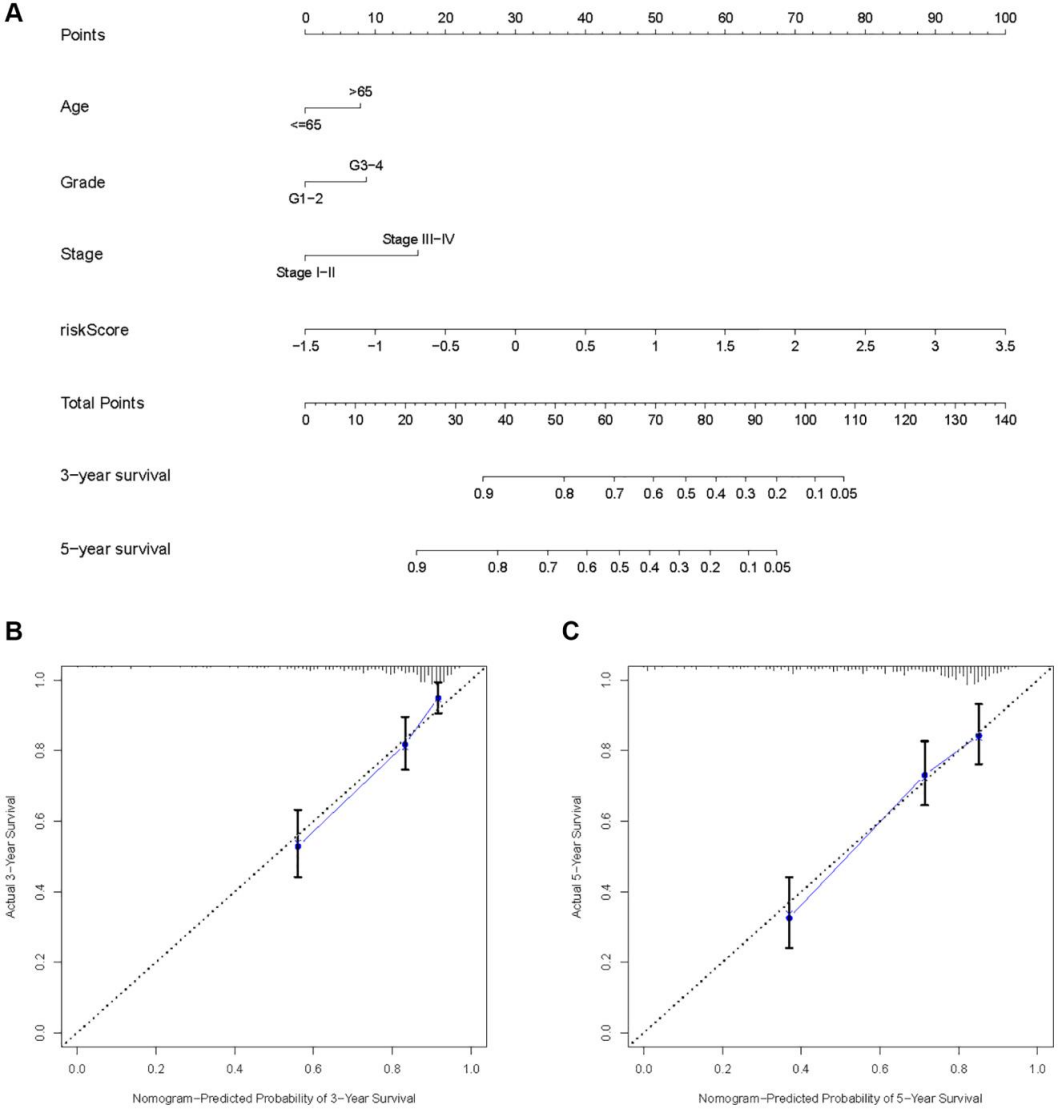
**Construction of a risk score-based prognostic model for KIRC.** (**A**) Construction of a risk score-based nomogram for predicting 3- and 5-year OS in KIRC samples. (**B**, **C**) Calibration curves to determine the performance of nomogram to predict 3- and 5-year OS in KIRC samples.

As a result of the univariate and multivariate analyses, independent prognostic factors of the KIRC samples were incorporated into the prognostic model. By transforming complex regression equations into visual graphs, nomograms make the results of predictive models more understandable [23]. A nomogram predicting the probability of 3- and 5-year OS was plotted on the basis of this advantage (Figure 11A). The concordance index (C-index) = 0.754 reflected the decent accuracy of the nomogram in predicting the 3- and 5-year OS of KIRC. The calibration curves further showed the consistency between the nomogram-predicted OS probabilities for the KIRC samples and the actual OS for the KIRC samples at 3- and 5-year (Figure 11B, 11C).

### Immune characteristics

**Figure 12:**
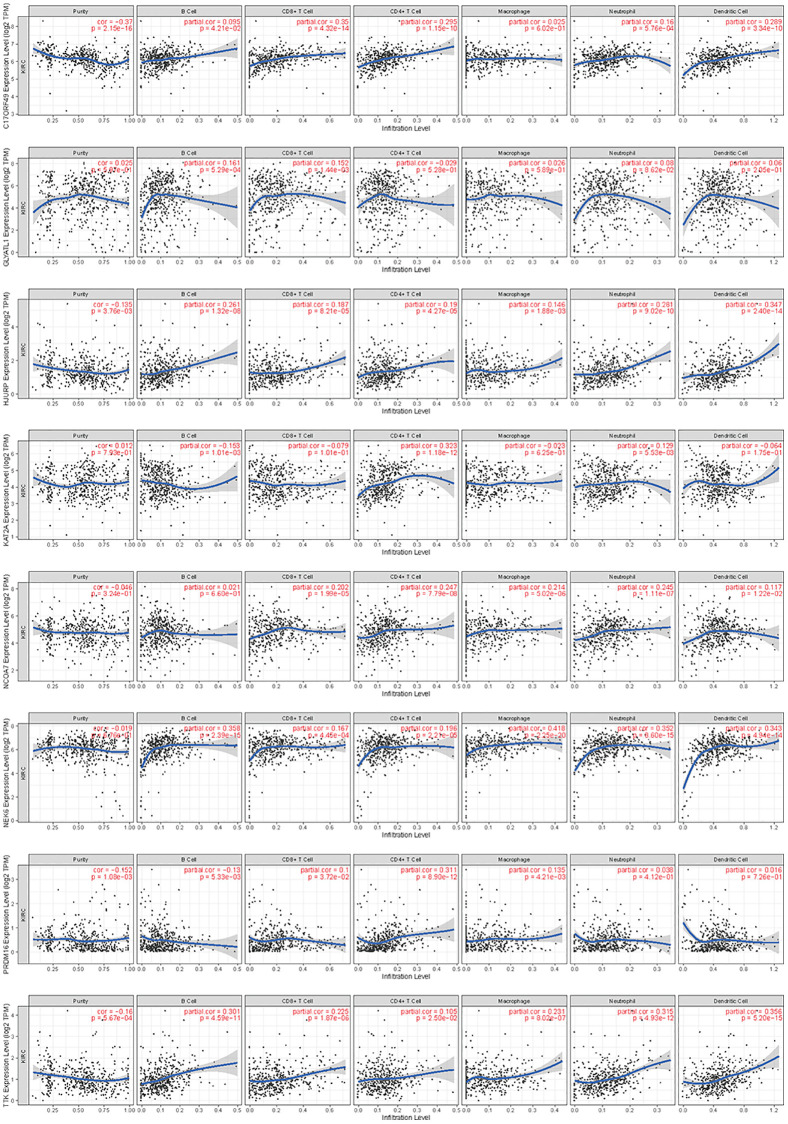
Correlation analysis of the expression of HMs with immune cell infiltration in TIMER database.

**Figure 13:**
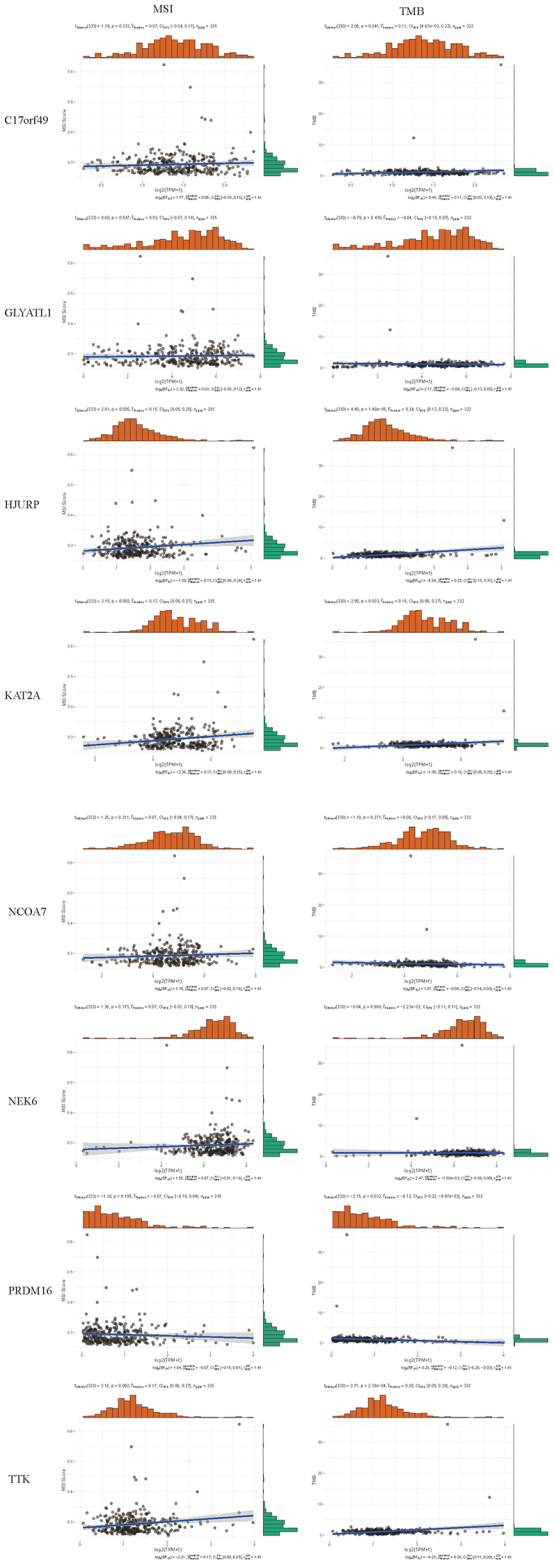
Correlation analysis of the expression of HMs with the level of MSI and TMB.

By searching the TIMER database, we found correlations between expression level of these 8 HMs (*C17orf49, GLYATL1, HJURP, KAT2A, NCOA7, NEK6, PRDM16, TTK*) and the infiltration level of six common immune cells (B Cell, CD8+ T Cell, CD4+ T Cell, Macrophage, Neutrophil and Dendritic Cell) (Figure 12). The expression of *C17orf49* closely correlated with the infiltration of CD8+ T Cell (cor = 0.35, *p* = 4.32e−14), CD4+ T Cell (cor = 0.295, *p* = 1.15e−10), Neutrophil (cor = 0.16, *p* = 5.76e−04), and Dendritic Cell (cor = 0.289, *p* = 3.34e−10). The expression of *GLYATL1* significantly correlated with the infiltration of B Cell (cor = 0.161, *p* = 5.29e−04) and CD8+ T Cell (cor = 0.152, *p* = 1.44e−03) only. *HJURP* showed a close association with immune infiltration, and its expression level significantly correlated with B Cells (cor = 0.261, *p* = 1.32e−08), CD8+ T Cell (cor = 0.187, *p* = 8.21e−05), CD4+ T Cell (cor = 0.19, *p* = 4.27e−05), Macrophage (cor = 0.146, *p* = 1.88e−03), Neutrophil (cor = 0.281, *p* = 9.02e−10) and Dendritic Cell (cor = 0.347, *p* = 2.40e−14). The expression level of *KAT2A* had significantly negative correlation with the infiltration level of B cells (cor = −0.153, *p* = 1.01e−03), however, it had significantly positive correlation with the infiltration level of CD4+ T cells (cor = 0.323, *p* = 1.18e−12) and Neutrophil (cor = 0.129, *p* = 5.53e−03). The expression of *NCOA7* closely correlated with infiltration of CD8+ T Cell (cor = 0.202, *p* = 1.99e−05), CD4+ T Cell (cor = 0.247, *p* = 7.79e−08), Macrophage (cor = 0.214, *p* = 5.02e−06), Neutrophil (cor = 0.245, *p* = 1.11e−07) and Dendritic Cell (cor = 0.117, *p* = 1.22e−02). Both *NEK6* and *TTK* show a close correlation with immune cell infiltration. The expression of *NEK6* and *TTK* closely and positively correlated with the infiltration of these six immune cells (B Cells, CD8+ T Cell, CD4+ T Cell, Macrophage, Neutrophil and Dendritic Cell) (*p* < 0.01). The expression of *PRDM16* had significantly negative correlation with the infiltration of B cells (cor = 0.13, *p* = 5.33e−03), but significantly positive correlation with the infiltration of CD8+ T Cell (cor = 0.1, *p* = 3.72e−02), CD4+ T Cell (cor = 0.311, *p* = 8.90e−12) and Macrophage (cor = 0.135, *p* = 4.21e−03). We further investigated the correlation between the expression of these 8 HMs and the level of MSI and TMB. The results showed that the expression of *C17orf49* significantly correlated with the level of TMB (r = 0.11, *p* = 0.041). The expression of *HJURP* significantly correlated with the level of MSI (r = 0.15, *p* = 0.005) and TMB (r = 0.24, *p* = 1.49e−05). *KAT2A* likewise showed a close association with MSI (r = 0.17, *p* = 0.002) and TMB (r = 0.16, *p* = 0.003). The expression level of *PRDM16* negatively correlated with the level of TMB (r = −0.12, *p* = 0.032). The expression level of *TTK* showed a meaningful correlation with the level of both MSI (r = 0.17, *p* = 0.002) and TMB (r = 0.20, *p* = 2.39e−04) (Figure 13).

**Figure 14:**
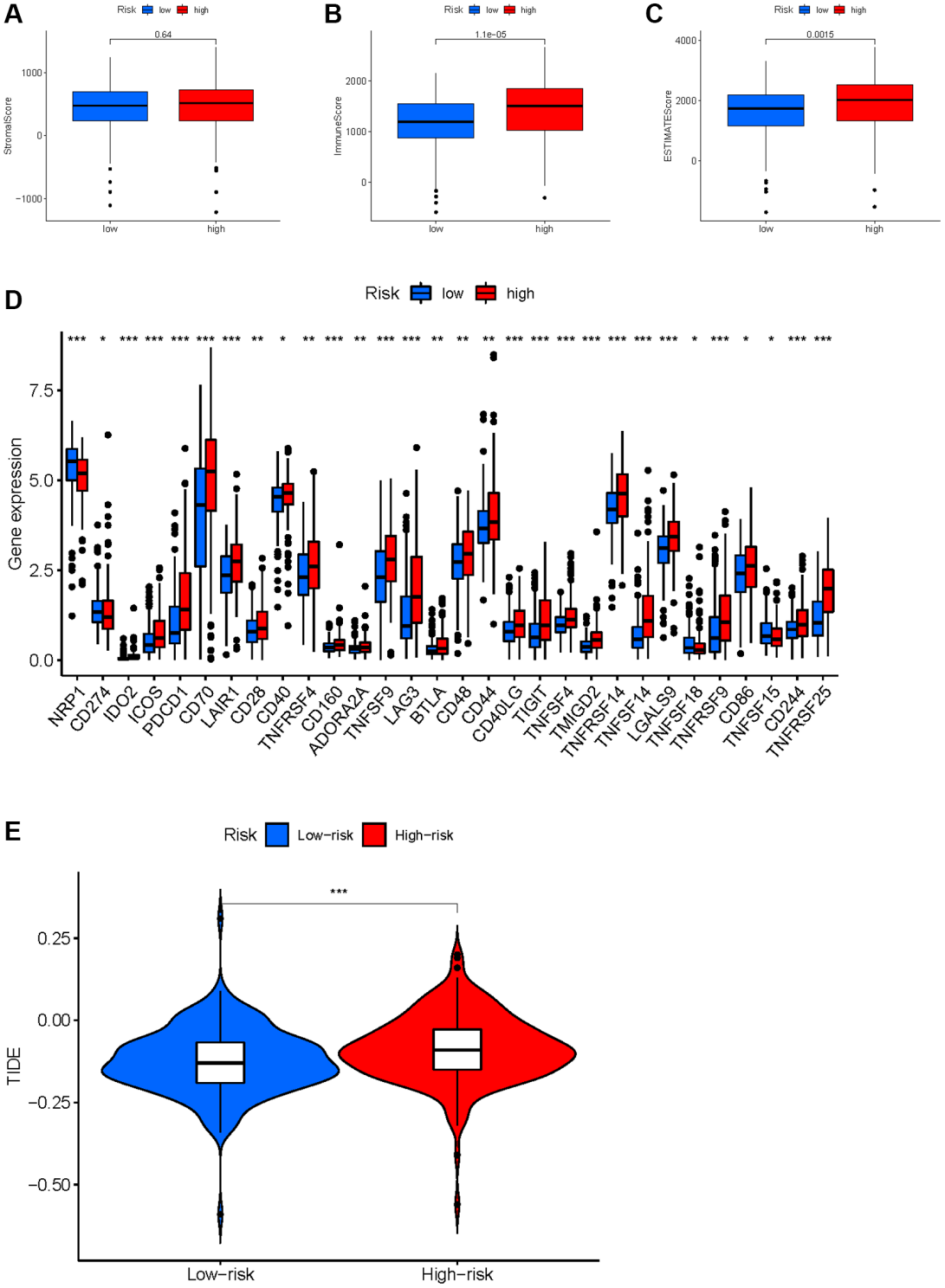
**Correlation between HMs-based signature and immune characteristics.** (**A**–**C**) Analysis of the tumour microenvironment between high- and low-risk groups by ESTIMATE algorithm. (**D**) Analysis of differences in immune checkpoint expression between high- and low-risk groups. (**E**) Analysis of differences in TIDE scores between high- and low-risk groups.

Based on the evidence that these HMs were associated with immunity, we analyzed the correlation between HMs-based signature and immune characteristics. Analysis of differences in the TME between high- and low-risk groups showed that the level of immune cell infiltration in the TME was significantly higher in the high-risk group than in the low-risk group (*p* = 1.1e−05) (Figure 14A–14C). We further analyzed the differences in the expression of immune checkpoints in KIRC samples between high- and low-risk groups, and result showed that the level of expression of *IDO2*, *ICOS*, *PDCD1*, *CD70*, *LAIR1*, *CD28*, *CD40*, *TNFRSF4*, *CD160*, *ADORA2A*, *TNFSF9*, *LAG3*, *BTLA*, *CD48*, *CD44*, *CD40LG*, *TIGIT*, *TNFSF4*, *TMIGD2*, *TNFRSF14*, *TNFSF14*, *LGALS9*, *TNFRSF9*, *CD86*, *CD244 and TNFRSF25* were significantly higher in the high-risk group than in the low-risk group (Figure 14D). In addition, a significant finding was that the TIDE scores of KIRC samples in the high-risk group were significantly higher than those of samples in the low-risk group (Figure 14E), revealing that KIRC in the high-risk group was more prone to immune escape.

### Drug sensitivity analysis

**Figure 15:**
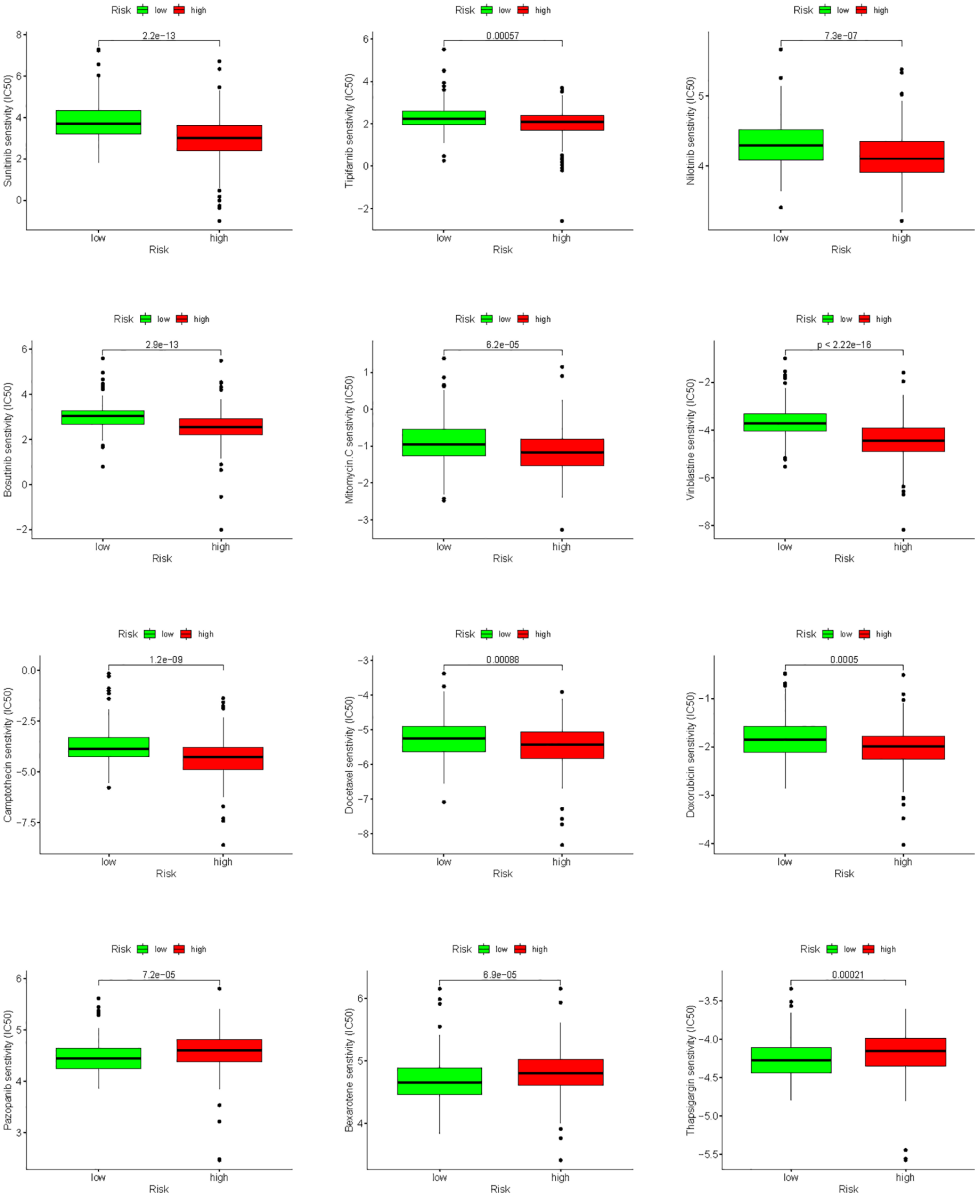
Analysis of differences in sensitivity to chemotherapy drugs between high- and low-risk groups.

To improve chemotherapy outcomes in KIRC patients, it is important to understand KIRC’s sensitivity to chemotherapy. Analyses of chemotherapy drug sensitivity in KIRC samples with high or low risk showed that KIRC samples with high risk had lower IC_50_ values for Sunitinib, Tipifarnib, Nilotinib, Bosutinib, Mitomycin. C, Vinblastine, Camptothecin, Docetaxel and Doxorubicin compared with KIRC samples with low risk (Figure 15), implying that KIRC samples with high risk were more sensitive to these nine chemotherapy drugs. In contrast to the high-risk group, the low-risk group had significantly lower IC_50_ values for Pazopanib, Bexarotene, and Thapsigargin in KIRC, implying that KIRC samples with low risk were more sensitive to these three chemotherapeutic drugs.

## DISCUSSION

HMs involved in histone modifications, which were important content of epigenetic [24, 25]. KIRC has been shown to be a cancer closely associated with epigenetic alterations [26–28]. Although studies have now demonstrated that HMs were associated with KIRC occurrence or progression [27, 29], comprehensive analysis of the predictive value of HMs in KIRC is lacking.

In the present study, we firstly obtained HMs from top journals, and then by differential analysis of mRNA expression in KIRC, we obtained 49 HMs that were differentially expressed in KIRC. The results of enrichment of GO and KEGG uncovered that HMs were closely involved in epigenetic regulation. In addition, consensus cluster analysis based on the expression of HMs revealed that the OS and clinical characteristics (grade, stage and gender) of the two clusters differed significantly. To evaluate whether HMs have prognostic value in the KIRC samples, we successfully constructed an HMs-based prognostic signature by Cox analysis and LASSO analysis, and the test and entire sets validated the good performance of the HMs-based signature in predicting KIRC sample’s prognosis. The results of the clinical characteristics analysis and subgroup survival analysis implied poor prognostic outcomes for KIRC patients in the high-risk group. Nomogram and calibration curves-based HMs signature revealed the satisfactory performance of a risk score in combination with traditional clinical variables in forecasting the prognosis of KIRC.

Of these 8 HMs used to construct prognostic signature, some have displayed significant associations with KIRC progression. *KAT2A* can drive the glycolytic process of KIRC and promote the progression of KIRC by activating *MCT1* [30]. *NEK6* is an oncogenic gene in KIRC that can be upregulated by LncRNA FAM13A-AS1 to promote the progression of KIRC [31, 32]. *PRDM16* can exert its tumor growth inhibitory effect by suppressing the expression of HIF-targeted gene in KIRC [33]. *TTK* can promote KIRC growth and metastasis by increasing the proliferative and invasive capacity of KIRC cells [34]. The functions of *C17orf49*, *GLYATL1, HJURP and NCOA7* in KIRC have not been reported. Our future studies will focus on these HMs.

Numerous studies have shown that tumor progression depends on TME [35–39], of which immune cells were important component and closely associated with tumor progression [40, 41]. The results in the TIMER database showed that all 8 HMs (*C17orf49*, *GLYATL1*, *HJURP*, *KAT2A*, *NCOA7*, *NEK6*, *PRDM16*, *TTK*) correlated significantly with cell infiltration by immune cells, especially *NEK6*, *TTK* and *HJURP*, which all correlated significantly with the infiltration of the 6 common immune cells. Furthermore, the results of correlation analyses of the expression of HMs constituting prognostic signature with the level of MSI and TMB indicated that the expression of *HJURP*, *KAT2A* and *TTK* closely correlated with MSI and TMB, and the expression of *C17orf49* and *PRDM16* significantly correlated with TMB. These findings implied that our prognostic signature may be closely related to the immune characteristics of KIRC. TME analysis revealed a greater proportion of immune cells in high-risk group than in low-risk group, uncovering that immune cell infiltration may contribute to a worse prognosis for high-risk KIRC patients than for low-risk KIRC patients. Based on the analysis of the immune checkpoint expression differences between patients in the high and risk groups, we can infer that patients in the high risk group were immunosuppressed. Immune checkpoint blockade is now becoming an influential therapeutic approach for several cancers, including KIRC, [42–45] and has shown remarkable results. However, there were still some patients who received unsatisfactory treatment outcomes, and one important reason for this is the immune escape [46–48]. We analyzed the likelihood of immune escape in groups of KIRC patients at high and low risk. KIRC patients in the high-risk group have higher TIDE scores than these in the low-risk group, uncovering that tumors in KIRC patients in the high-risk group were more likely to undergo immune escape. Therefore, it triggers a reflection that the factor of possible immune escape needs to be taken into account when immunotherapy of KIRC samples is not effective. Results of sensitivity analysis of KIRC samples to chemotherapy drugs uncovered that high-risk KIRC patients benefit more from Sunitinib, Tipifarnib, Nilotinib, Bosutinib, Mitomycin. C, Vinblastine, Camptothecin, Docetaxel and Doxorubicin than low-risk patients, while KIRC patients in the low-risk group benefited more from the use of Pazopanib, Bexarotene, and Thapsigargin than those in the high-risk group. This has led to a consideration that we need to individualize the drug therapy in KIRC samples according to their risk level.

There were some limitations in our research. The lack of prognostic data on KIRC patients in the Gene Expression Omnibus (GEO, https://www.ncbi.nlm.nih.gov/geo/) database [49] and the fact that the International Cancer Genome Consortium (ICGC, https://dcc.icgc.org/) database [50] only contained patient data on KIRC patients derived from TCGA, prevented us from validating the results of this study using an independent dataset. This may have led to some selection bias. In addition, cell and animal experiments are needed to further mine the latent function of HMs signature in KIRC. Thus, our findings in this study will be further validated in future studies.

## CONCLUSION

We successfully established and validated a risk model to predict the prognosis, immune characteristics and sensitivity to chemotherapy drugs in KIRC samples based on 8 HMs (*C17orf49*, *GLYATL1*, *HJURP*, *KAT2A*, *NCOA7*, *NEK6*, *PRDM16*, *TTK*), which may have potential for application in the management of KIRC patients.
